# Efficient Detection of West Nile Virus in Urine Specimens by a Novel In-House RT-qPCR Detection Kit

**DOI:** 10.1155/cjid/6513971

**Published:** 2025-01-07

**Authors:** Gülten Tuncel, Gökçe Akan, Melis Kalaycı, Buket Baddal, Ayşegül Bostancı, Kaya Suer, Cenk Serhan Özverel, Tamer Şanlıdağ

**Affiliations:** ^1^DESAM Research Institute, Near East University, Nicosia, Cyprus; ^2^Biochemistry Department, MUHAS Genetics Laboratory, School of Medicine, Muhimbili University of Health and Allied Sciences, Dar es Salaam, Tanzania; ^3^Department of Medical Microbiology and Clinical Microbiology, Faculty of Medicine, Near East University, Nicosia, Cyprus; ^4^Department of Infectious Diseases and Clinical Microbiology, Faculty of Medicine, Near East University, Nicosia, Cyprus

**Keywords:** rapid diagnosis, real-time PCR detection, urine, West Nile Virus

## Abstract

West Nile Virus (WNV) infection represents a major global public health challenge. Even though most of the patients are asymptomatic, some cases progress to critical condition which may be fatal. Diagnosis traditionally relies on serological methods, but their limitations, including cross-reactivity, highlight the need for alternative approaches. Here, we present the development and validation of a novel RT-qPCR assay for precise and rapid detection of WNV RNA in urine, emerging as a promising specimen due to its noninvasive collection and high viral load. The assay demonstrates high efficiency and sensitivity, with a detection limit comparable to commercially available kits. This study highlights the importance of in-house kit design as a diagnostic tool in regions affected by emerging tropical infections, such as WNV, exemplified Cyprus. It emphasizes the critical role of low-cost, early detection with high sensitivity and specificity in infection control and surveillance efforts.

## 1. Introduction

West Nile Virus (WNV) has emerged as a global public health concern since its initial identification in the West Nile region of Uganda in 1937 [1]. Over the decades, WNV has traversed continents, displaying a remarkable capacity for adaptation and persistence in diverse ecological settings, and has caused the onset of multiple pandemics over the years in different regions such as Greece, Israel, Romania, and the USA [2, 3]. WNV is an arbovirus that spreads to humans through mosquito bites. The critical point in spreading WNV to diverse geographical regions is that the WNV-infected birds serve as viral reservoirs and mosquitoes may get the infection by feeding on these infected birds [4]. In this context, bird migration can significantly contribute to WNV spread. However, it is also important to note that although most human cases arise from mosquito bites, direct transmission between humans has been reported through organ transplants, blood transfusions or exposure during pregnancy [5, 6].

WNV is a member of the Flaviviridae family and is a single-stranded RNA virus with a spherical shape, surrounded by a lipid-bilayer envelope [7]. Glycoproteins located on the envelope act as protruding spikes, mediating the attachment of the virus to the host cells. RNA translation from the 11 kb-long WNV genome results in the synthesis of three structural (capsid, envelope, and membrane) and seven nonstructural (NS1, NS2A, NS2B, NS3, NS4A, NS4B, and NS5) proteins [8]. While structural proteins are essential for virus entry and encapsulation of the viral genome, nonstructural proteins are vital to form the replication complex [9]. Among these, the viral RNA-dependent RNA polymerase (NS5) is highly conserved and was shown to be associated with interferon (IFN) resistance among its critical involvement in the viral replication process and is recognized as a virulence determinant for WNV in mammalian hosts [10–12].

Most WNV infections in humans are asymptomatic. The cases of symptomatic West Nile disease, which usually begins with a sudden headache, can progress with muscle pain, weakness, loss of appetite, nausea, vomiting, and rash. Parkinson's-like tremor symptoms can also be observed in patients who suffer from the disease severely [8, 13, 14]. According to a study conducted in America, it has been stated that 20% of the infected patients develop self-limited fever [15]. In addition to the potential complications of high fever, there is a risk of meningitis, encephalitis, acute jaundice, and stroke in severe forms of infection due to neuroinvasion. Furthermore, neurological anomalies, cranial nerve anomalies, and seizures may also develop [13]. The mortality rate of individuals infected with WNV has been reported to be 3%–15% [16]. Elderly and immunocompromised persons are at increased risk for the development of neuroinvasive West Nile disease [12].

The diagnosis of a probable case of WNV disease can be established by the detection of virus-specific IgM antibodies in the cerebral spinal fluid (CSF) or serum [8]. WNV-specific IgM antibodies can be detected no earlier than 3 days and no later than 90 days after the onset of the disease. Therefore, as the CDC notes, positive IgM antibodies may also reflect a past infection. Overall, as the immune response against WNV is quite variable, it is important to note that the only robust feature is the IgG seroconversion, which indicates a past infection. According to the CDC, the laboratory criteria for confirming acute WNV infection include several pivotal indicators. These include isolating the virus or detecting specific viral antigens or nucleic acids in tissue, blood, CSF, or other body fluids. Additionally, a fourfold or higher change in virus-specific antibody titers in paired serum samples is considered a significant finding. The presence of virus-specific IgM antibodies in serum, alongside virus-specific neutralizing antibodies in the same or a subsequent specimen, also supports the diagnosis. Furthermore, the detection and characterization of virus-specific IgM antibodies in CSF, accompanied by a negative result for other arbovirus-specific IgM antibodies in the CSF from the region where exposure occurred, is another crucial criterion for confirmation [17].

Given the absence of vaccine or treatment regime available for WNV, which is listed as a global threat by the CDC, providing fast and accurate results is vital, particularly for infected individuals who are elderly and with low immunity [18]. Here, we introduce the development of a rapid, low-cost in-house RT-qPCR assay kit that can detect the presence of WNV nucleic acid in urine samples of infected individuals within the first 2 days of infection with high sensitivity.

## 2. Materials and Methods

### 2.1. WNV Genome Sequences

Complete or partial genome sequences that were submitted to the NCBI Nucleotide database between January 2011 and September 2023, covering African and European countries, particularly in the Mediterranean region, were analyzed to determine highly conserved regions in the WNV genome. Selected reference sequences from both WNV lineage 1 and lineage 2 (GenBank reference numbers HQ596519.1, MW627239.1, MW751846.1 and OR091152.1) were aligned by Basic Local Alignment Search Tool for nucleotide sequences (blastn) by NCBI and MAFFT software with default settings. The consensus sequences (100% alignments) within the NS5 gene region were selected for primer and probe design.

### 2.2. Primer Probe Design

Upon determination of conserved regions, Primer Blast Tool by NCBI, SnapGene and Primer3 were used for sequence-specific primer and probe design. The human RP gene was used as the housekeeping gene and gene-specific primers and probes were designed to target RP as an internal control. Gene-specific probes were labelled with different fluorescent dyes. Fluorescein amidites (FAM) for the WNV NS5 conserved region and hexachloro-fluorescein (HEX) for the human RP gene were used. Specificity tests of designed primer and probe sequences were performed with in silico tools using 27 different viral and bacterial strains. Other flaviviruses and arboviruses such as Zika virus, Dengue Yellow Fever virus and Chikungunya virus were specifically included in the in silico specificity tests to avoid any possible cross-reaction during PCR.

### 2.3. RT-qPCR Conditions and Primer Probe Optimization

Primer and probe optimizations were performed using Amplirun WNV RNA Control (Cat #MBC069-R, AmpliRun, Vircell). Upon determination of the optimal primer annealing temperature, primer and probe concentrations and cycling conditions were optimized in Rotor-Gene Q Plex Real-Time PCR (Qiagen). The reaction mix was prepared with 10 *μ*L Takyon One-Step Low Rox Probe 5X MasterMix dTTP (Cat #UF-LP5X-RT0501, Takyon, Eurogentec), 0.4 *μ*M final primer concentration, 0.2 *μ*M final probe concentration, 5 *μ*L RNA template and RNase/DNase-free ddH_2_O up to 20 *μ*L. Cycling conditions used were as follows; reverse transcription at 45°C for 20 min, initial denaturation at 95°C for 3 min, 45 cycles of denaturation at 95°C for 10 s and amplification at 59°C for 40 s. Fluorescence reading was performed at the amplification step for FAM and HEX channels.

### 2.4. Sample Collection and Nucleic Acid Isolation

Serum, CSF, and urine samples were collected from patients who were admitted to Near East University Hospital, Nicosia, Northern Cyprus in October 2023 with symptoms consistent with WNV infection. Biochemical and serological analyses were performed with the samples obtained from the patients. Results are represented in Table 1.

RNA isolation was performed using GeneAll Ribospin vRD II (Cat #322-150, GeneAll Biotechnology) following the manufacturer's instructions with 30 *μ*L final elution volume. 5 *μ*L of isolated RNA was used as a template for the RT-qPCR experiments. Consent forms were signed by the patients involved in the study.

### 2.5. Amplification Efficiency and Analytical Sensitivity

A ten-fold dilution series of WNV RNA Control (Amplirun, Cat #MBC069-R) with known concentration (viral copy number/*μ*L) was prepared (5 × 10^4^, 5 × 10^3^, 5 × 10^2^, 5 × 10^1^ copies/reaction) and used as a template in the real-time PCR assays under optimized conditions. The slope calculation was conducted using linear regression to determine the PCR (E), correlation coefficient (*R*^2^), and sensitivity. A standard curve was subsequently generated based on the results. Moreover, 20 replicates of the 10-fold dilution series (1 × 10^−2^, 1 × 10^−1^, 1, 1 × 10^1^, 1 × 10^2^, 1 × 10^3^, and 1 × 10^4^) were performed for calculations of 95% limit-of-detection (LOD 95) as copies/reaction using the Probit regression analysis using SPSS (IBM, version 24).

### 2.6. Diagnostic Performance

Validation of the kit performance was performed by using the commercially available kit Sacace WNV Real-TM (Cat #V53-50FRT, Sacace Biotechnologies, Como, Italy) PCR kit according to the manufacturer's instructions. Two clinically confirmed WNV positive samples, three WNV negative samples, and their 1:1 dilution were analyzed, in the same PCR machine (Rotor-Gene Q Series Software (Qiagen)) with the same consumables. The results from both methods were analyzed and compared.

### 2.7. Data Analysis

Amplification curves of human internal controls and viral genes were assessed to evaluate the results. The threshold was automatically adjusted by the Rotor-Gene Q Series Software (Qiagen), and cycle threshold values were obtained accordingly. The positive cut-off value was set at cycle threshold number ≤  37 with a sigmoidal curve. Positive control samples were examined, runs where the positive control met the criteria were considered valid and any patient samples meeting the criteria were classified as positive.

### 2.8. Ethical Approval

The study received approval from the Institutional Ethical Review Board of Near East University with an IRB number of YDU/2024/120-1817.

## 3. Results

### 3.1. Standardization of the RT-qPCR Conditions

The multiplex RT-qPCR assay was optimized for WNV diagnosis, simultaneously targeting a conserved region of the WNV genome (NS5 gene region), and the human RP gene as the internal control. Upon primer and probe optimization with a commercially available WNV control sample (Cat #MBC069-R, AmpliRun, Vircell), the assay was evaluated using CSF, serum and urine samples obtained from five patients that were admitted to Near East University Hospital, Nicosia, Northern Cyprus in October 2023 with symptoms associated with WNV. RNA samples obtained from CSF, serum, and urine samples were tested with the RT-qPCR assay in optimized conditions (Table 1). The presence of WNV nucleic acid was detected in the clinical samples of two patients. All patient samples were simultaneously tested for WNV IgM and IgG, and the results were found to be in concordance with the RT-qPCR results.

**Figure 1:**
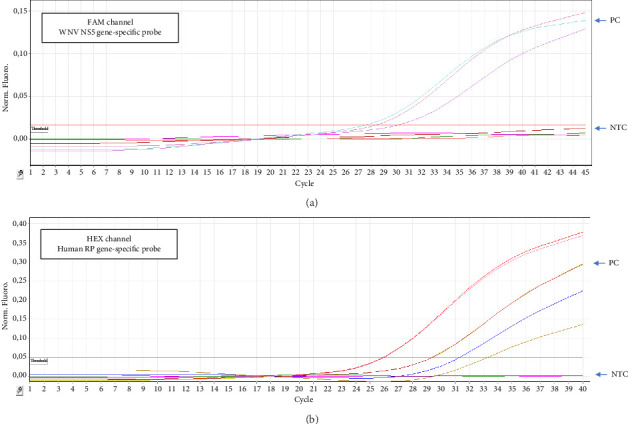
RT-qPCR amplification curve graphs of positive control (PC), no template control (NTC), and patient samples for (a) WNV NS5 gene-specific region and (b) human RP gene-specific region are represented in the graph.

Detection of WNV nucleic acid in the urine samples of the infected individuals was more efficient compared to the CSF samples of the same patients. Overall, viral RNA was not detected in serum samples. A commercial WNV control sample (Cat #MBC069-R, AmpliRun, Vircell) was used as a positive control in all reactions. In WNV positive samples and positive control samples, RP (human internal control) and WNV target region were amplified simultaneously forming sigmoidal curves. In WNV-negative samples, RP was the only gene amplified with a sigmoidal amplification curve. In the negative control reactions, ddH_2_O was used as the template, which led to no amplification line (Figure 1).

### 3.2. Assay Validation

For assay validation, a commercially available Sacace WNV Real-TM (Cat #V53-50FRT, Sacace Biotechnologies, Como, Italy) kit was used. Two clinically confirmed WNV-positive patient samples, three WNV-negative patient samples and their 1:1 dilutions, positive control, and negative control samples were analyzed simultaneously with both kits following the manufacturer's instructions. The in-house kit exhibited 100% positive percent agreement with the commercially available diagnostic kit in urine samples. Moreover, the CSF sample detected to be positive with the in-house kit was not detected with the commercial kit.

### 3.3. RT-qPCR Efficiency and LOD

**Figure 2:**
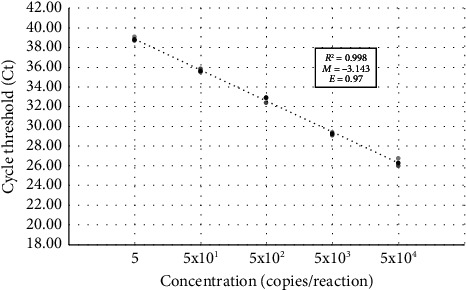
Standard curve analysis for multiplex RT-qPCR of WNV NS5 gene primers. The template RNA was serially diluted with a range of 5 × 100^−5^ × 10^4^ copies/reaction. The reactions were carried out in triplicate. The amplification efficiency (E) is shown on the graph.

The standard curve analysis was performed to evaluate the assay's efficiency, sensitivity, and LOD. A dilution series was prepared with the positive control with a dilution factor range of 1 to 10^4^, where the undiluted sample contained 10,000 copies/*μ*L. Triplicate RT-qPCR analysis revealed that the results were consistent across technical replicates. The RT-qPCR efficiency for the WNV target gene was 0.97, and the *R*^2^ value was 0.993, which indicated the consistency and reliability of the assay (Figure 2).

**Figure 3:**
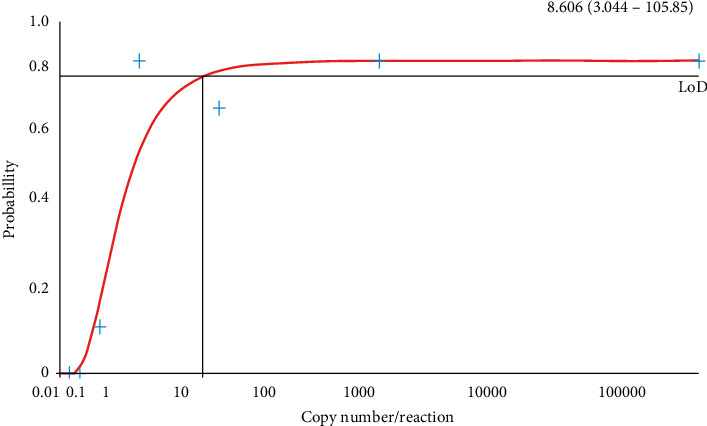
Determination of the limit of detection 95% (LOD 95) for WNV-NS5 primers. The probit regression analysis used for the determination of LODs is based on twenty technical replicates of 10-fold dilution series (1 × 10^−2^, 1 × 10^−1^, 1, 1 × 10^1^, 1 × 10^2^, 1 × 10^3^, and 1 × 10^4^) of synthetic WNV RNA.

For the analysis of Probit regression, 20 technical replicates of 10-fold dilution series (1 × 10^−2^, 1 × 10^−1^, 1, 1 × 10^1^, 1 × 10^2^, 1 × 10^3^, and 1 × 10^4^) of control WNV RNA were used, and the LOD 95 of WNV-NS5 gene was determined. The LOD 95 of the WNV-NS5 gene was 8.606 copies/reaction, and estimated 95% confidence interval (CI) for the gene was between 3.044 and 105.85 copies/reaction (Figure 3).

## 4. Discussion

WNV infection in humans has been linked to two lineages of the WNV, namely lineage 1 (WNV-1) and lineage 2 (WNV-2). Various strains of WNV-1 have been circulating in Europe and the Mediterranean basin, leading to human outbreaks since at least the late 1950s. On the other hand, WNV-2 primarily comprises isolates from sub-Saharan Africa, although some WNV-2 strains have been found in humans and mosquitoes beyond the African continent [19–21]. Although the disease typically presents as asymptomatic in endemic regions such as sub-Saharan Africa and the Middle East, it manifests through outbreaks with varying degrees of neurological damage in other parts of the world [20]. Even though the first reported case in Cyprus dates back to August 2016, there were no reported cases from Northern Cyprus until 2022 [22, 23]. It is believed that one possible reason for the recent emergence of previously undetected tropical diseases on the island is the increased migration, particularly from the African continent. This trend is expected to continue growing in the coming years [24].

The initial step in diagnosing WNV infection is the identification of WNV-specific antibodies using immune-enzymatic techniques. Serologically positive cases are subsequently validated by the detection of the virus in clinical samples [20]. The serological diagnosis relies on identifying IgM and IgG antibodies against WNV, however, the primary limitation hindering the clinical significance of serological methods is the extensive antigenic cross-reactivity observed among all flaviviruses [25]. Amino acid sequences of M and NS proteins are more conserved across flaviviruses, contributing to the cross-reactivity issues [21]. In some cases, the persistence of IgM antibodies can prevent the diagnosis of ongoing infection [26]. The confirmation of ongoing infection can be achieved through a virus-neutralizing test (VNT) or by detecting the virus genome using real-time PCR [20]. Although VNT against WNV is considered the gold standard due to its high specificity and ability to detect and quantify neutralizing antibodies, its drawback is the time-consuming nature of the assay and its relatively high cost. On the other hand, real-time PCR has become a rapid and exceptionally reliable diagnostic tool, notable for its high sensitivity, specificity, and low cost. Its prevalent use is evident in the diagnosis of viral infections, with WNV being among the commonly targeted viruses [25]. Therefore, PCR-based WNV diagnosis emerges as a significant alternative to serological methods, particularly in the early stages of the disease. In the present study, a novel in-house RT-qPCR assay targeting WNV lineage 1 and lineage 2 was developed for the rapid detection of WNV from clinical samples including blood, CSF, and urine. As represented in Table 1 and experienced during the optimization protocol, WNV RNA was successfully and more effectively detected in the urine specimens. WNV may demonstrate a particular tropism for renal tissues or manifest enhanced replicative capacity within the kidneys, consequently resulting in elevated viral particle concentrations in the urine compared to CSF and blood samples [27]. By molecular testing, the presence of WNV RNA in urine can be detected after 2 days of the symptoms, and according to previous studies, it is detectable for up to 1 month after the symptom onset, indicating that the successful isolation of WNV from urine is more effective during the initial days of infection [28, 29]. Moreover, CSF collection entails an invasive procedure known as a lumbar puncture or spinal tap. This procedure carries inherent risks and discomfort for the patient, necessitating specialized expertise for proper execution. Conversely, urine collection poses no such risks or likelihood of discomfort for the patient. Rapid and accurate detection of patients with an acute infection is crucial for patient isolation and prevention of transmission. Recent studies have indicated that patients with an acute infection exhibit elevated viral load and prolonged viral shedding in their urine samples [29]. Previous studies have indicated that detecting WNV RNA in urine is more efficient than in CSF in for probable cases [30–32]. For example, Barzon and his colleagues illustrated that utilizing real-time RT-PCR for the detection of WNV RNA in urine exhibited higher sensitivity compared to testing plasma and CSF in patients presenting with symptomatic WNV infection [30]. This highlights a potential diagnostic significance and emphasizes the importance of exploring urinary samples as a valuable source for the early detection of WNV infections.

The consistency and reliability of the current assay were represented by the standard curve analysis. RT-qPCR efficiency of the dilution series of the standards was 0.97 for the WNV NS5 gene, which matches the criteria for an efficient real-time PCR assay. The current protocol allows the diagnosis of WNV RNA with a LOD value of 8.606 copies/reaction for the WNV NS5 gene. The estimated 95% CI for the gene was calculated to be between 3.044 and 105.85 copies/reaction. Detection limits reported in the literature range between 15 and 40 copies per reaction, supporting the suitability of the present assay for diagnostic purposes [33, 34]. A comparative assessment was carried out between the designed kit and a commercially available diagnostic kit (Sacace-WNV Real-TM, Cat #V53-50FRT, Sacace Biotechnologies, Como, Italy) for validation. Two clinically confirmed WNV-positive patient samples, three WNV-negative patient samples and their 1:1 dilutions, positive control, and negative control samples were analysed with both kits, and results indicated a complete agreement, affirming the reliability of the custom-designed kit. In addition, the designed RT-qPCR assay has 127-min run-time that provides faster results compared to the commercial kits available on the market. Before the development of this assay, suspected patient samples from Northern Cyprus were routinely dispatched abroad for serology testing, resulting in prolonged periods of waiting time for test results. This delay adversely affected patients, sometimes culminating in fatalities before obtaining the diagnostic result. The introduction of this novel kit addresses this issue by providing rapid diagnostic outcomes within hours, thereby significantly reducing the risk of patient mortality.

In conclusion, the diagnostic assay described in this study represents the first WNV RT-qPCR diagnostic test kit developed in Cyprus. Our developed RT-PCR kit for the detection of the WNV presents a significant advancement in diagnostic technology with global implications. While our initial focus was to cater to the specific needs of Northern Cyprus, it is crucial to emphasize that the utility of this kit extends far beyond regional boundaries. With the increasing migrations and the rise of international travel, infectious diseases are unfettered. Furthermore, there is a growing concern among the global health community regarding the emergence of vector-borne diseases and their potential to fuel future pandemics [35, 36]. Given the versatility of our kit, which targets conserved regions of the WNV genome, it holds promise for widespread adoption in diverse geographical areas. However, due to the small population of Cyprus and the recent emergence of tropical infections such as WNV on the island, the assay could only be tested on a limited number of biological samples and it was not possible to carry out in vitro cross-reactivity tests. Another limitation of the study was that the viral stocks derived from cell cultures could not be used; instead commercially available purified RNA of WNV with known copy numbers was used.

Nevertheless, we are prepared for potential future pandemics characterized by vector-borne transmission, and the importance of robust diagnostic tools like our RT-PCR kit cannot be overstated. By enabling rapid and accurate detection of the WNV, our kit empowers healthcare professionals worldwide to swiftly respond to outbreaks and implement effective control measures, thereby ensuring protection of public health worldwide [37].

## Figures and Tables

**Table 1:** Representation of biochemical analysis, IgM/IgG data, and RT-qPCR results of patients with the designed kit.

Patient	CSF biochemical test results			IgM/IgG results		In-house RT-qPCR kit results		
	CSF glucose level (mg/dL)	CSF protein level (mg/dL)	CSF cell count (×10^3^/*µ*L)	WNV IgM	WNV IgG	CSF WNV PCR	Serum WNV PCR	Urine WNV PCR
1	79 (high)	38.3 (normal)	None	Positive	Positive	Negative	Negative	Negative
2	95.0 (high)	124 (high)	1820	Positive	Negative	**Positive**	Negative	**Positive**
3	98.7 (high)	71 (high)	250	Positive	Positive	Negative	Negative	Negative
4	215.1 (high)	44 (normal)	N/A	Positive	Negative	Negative	Negative	Negative
5	156.3 (high)	75 (high)	0.069	Positive	Positive	Negative	Negative	**Positive**

*Note:* Bold values signify the samples that WNV nucleic acid was detected with the designed RT-qPCR kit.

## Data Availability

The data that support the findings of this study are available from the corresponding author upon reasonable request.
